# Supplementary material for: Human angiotensin-converting enzyme 2 transgenic mice infected with SARS-CoV-2 develop severe and fatal respiratory disease
Source: JCI Insight. 2020 Oct 2;5(19):e142032. doi: 10.1172/jci.insight.142032 (PMC7566707; doi:10.1172/jci.insight.142032)
Supplement: Supplemental data [file jciinsight-5-142032-s155.pdf]

Supplementary Materials for

Human angiotensin-converting enzyme 2 transgenic mice infected with SARS-CoV-2 develop severe and fatal respiratory disease

Joseph W. Golden*, Curtis R. Cline, Xiankun Zeng, Aura R. Garrison, Brian D. Carey, Eric M. Mucker, Lauren E. White, Joshua D. Shamblin, Rebecca L. Brocato, Jun Liu, April M. Babka, Hypaitia B. Rauch, Jeffrey M. Smith, Bradley S. Hollidge, Collin Fitzpatrick, Catherine V. Badger and Jay W. Hooper*

*Corresponding author. E-mail: joseph.w.golden.ctr@mail.mil or jay.w.hooper.civ@mail.mil

This PDF file includes:
Supplemental Materials and Methods
Figs. S1 to S8
Tables S1

Supplemental Materials and Methods

Duplex *In situ* hybridization. Duplex in situ hybridization was performed using the RNAscope 2.5 HD Duplex Assay kit (Advanced Cell Diagnostics) according to the manufacturer's instructions with minor modifications. In addition to SARS-CoV-2 genomic RNA probe mentioned above (#854841, green), another probe with C2 channel (#848031-C2, red) specifically targeting human ACE2 (NM_021804.3) was designed and synthesized by Advanced Cell Diagnostics. ISH signal was amplified using kit-provided Pre-amplifiers and Amplifiers conjugated to either alkaline phosphatase or horseradish peroxidase, and incubated sequentially with a Fast Red and green chromogenic substrate solution for 10 min at room temperature. Sections were then stained with hematoxylin, air-dried, and coverslipped.

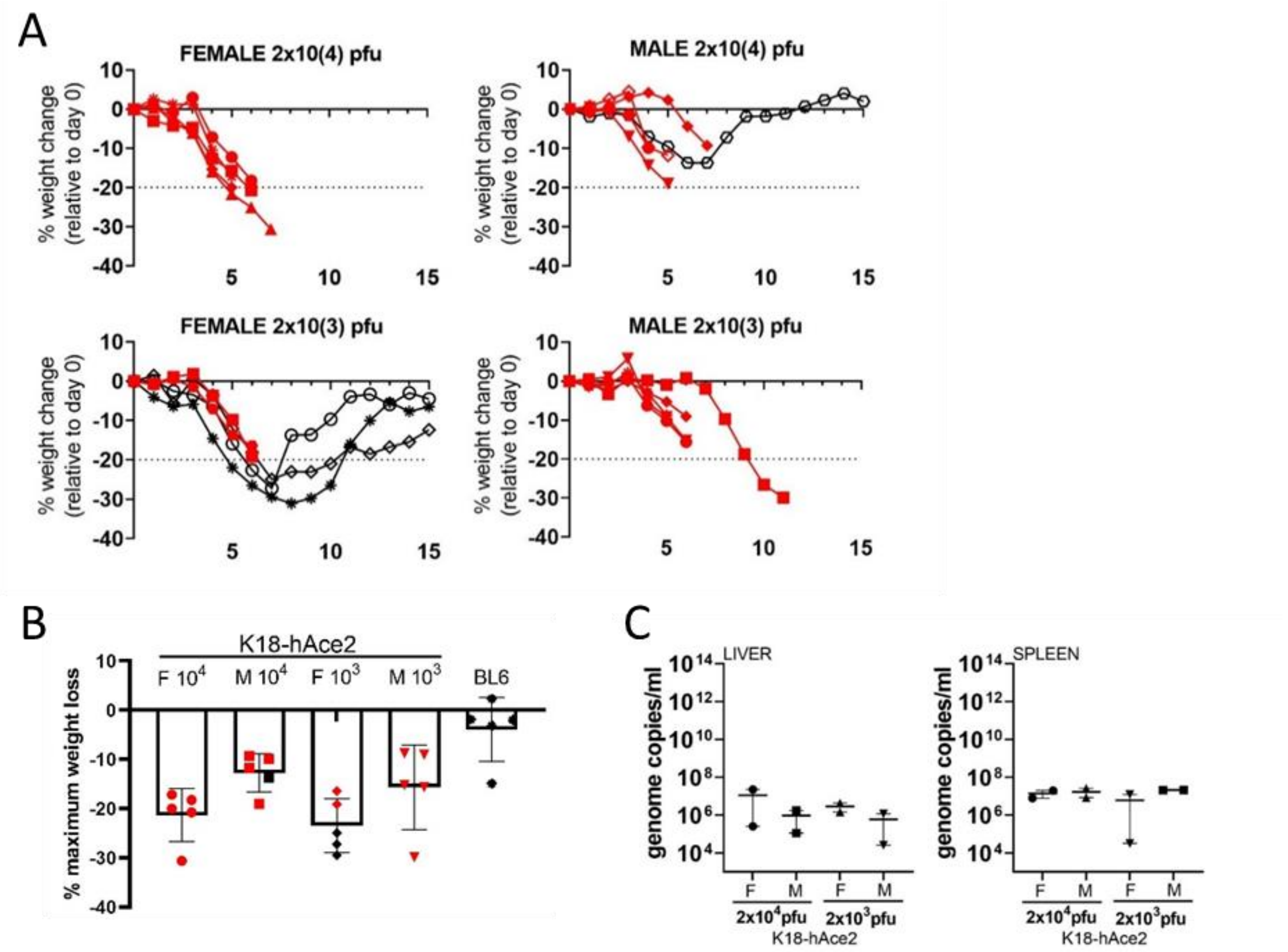

Figure S1. SARS-CoV-2 infection in K18-hAce2 transgenic mice. A. Individual weight loss in each challenge group is shown up to day 15. Red indicates an animal that died or was euthanized. **B**. Maximal weight loss over 15 day in infected mice. Red indicates mice that succumbed to disease. **C**. Titers in liver and spleen (n=2 mice/group) were examined on day 3 by qRT-PCR. Mean titers +/- SEM of the genome copies/ml were graphed.

Table S1: Summary of microscopic histopathology findings

Organ	Microscopic Finding	n	Severity*
Lung	Vasculitis in small to intermediate size vessels	21/22	minimal-moderate
	Alveolar septal inflammation / thickening	20/22	minimal-moderate
	Perivascular inflammation (in vessels without vasculitis)	15/22	minimal-moderate
	Alveolar accumulation of mononuclear leukocytes	10/22	minimal-mild
	Alveolar exudate (fibrin, edema)	9/22	minimal-moderate
	Type II pneumocyte hyperplasia	8/22	minimal-mild
	Multinucleate cells (macrophages or viral syncytia)	1/22	minimal
	Fibrin thrombi	1/22	moderate
	Positive In situ hybridization (ISH) for SARS-CoV-2	19/22	mild-severe
Nasal Turbinates	Degeneration, atrophy or erosion of olfactory epithelium	9/22	minimal-mild
	Exudate within nasal meatus	2/22	minimal
	Positive In situ hybridization (ISH) for SARS-CoV-2	19/22	minimal
Brain	Vasculitis (and/or perivascular inflammation) in small to intermediate sized vessels	14/22	minimal-moderate
	Microgliosis surrounding/adjacent to vessels	12/22	minimal-moderate
	Meningitis	8/22	minimal-moderate
	Necrosis	5/22	minimal-mild
	Fibrin thrombi	2/22	minimal
	Perivascular hemorrhage	1/22	mild
	Microgliosis, gray matter, multifocal	1/22	moderate
	Positive In situ hybridization (ISH) for SARS-CoV-2 (note 11/15 positive in the olfactory bulb)	15/22	minimal-marked

*Severity scores for ISH and histologic findings are based on the following: Minimal = If 10% or less of the cells in the section are ISH-positive or are affected respectively; Mild = If between 11% and 25% of the cells in the section are ISH-positive or are affected respectively; Moderate = If between 26% and 50% of the cells in the section are ISH-positive or are affected respectively; Marked = If between 51% and 79 of the cells in the section are ISH-positive or are affected respectively; Severe = If between 80% or more the cells in the section are ISH-positive or are affected respectively.

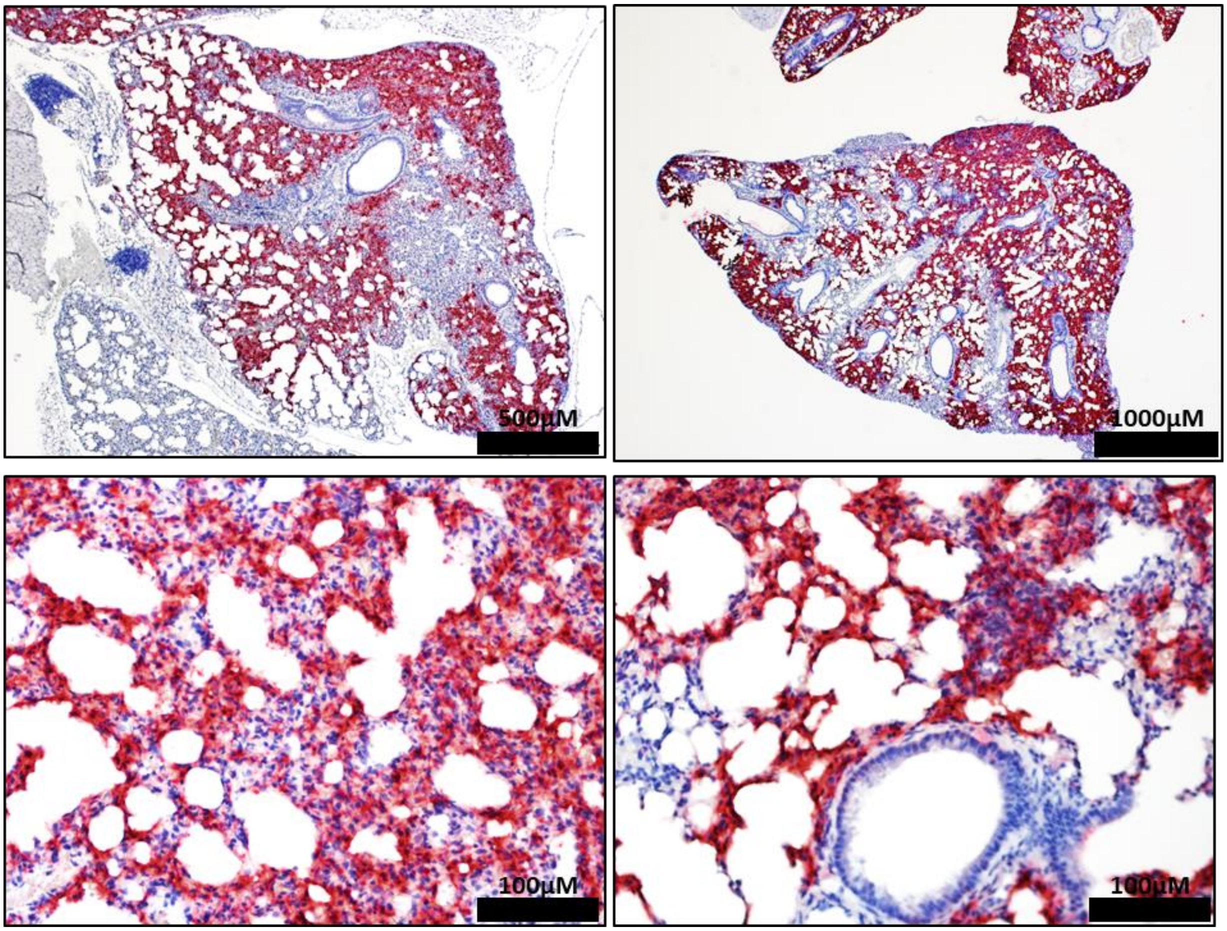

Figure S2. Infection of SARS-CoV-2 in the lungs in K18-Ace2 transgenic mice. Representative ISH images showing the presence of SARS-CoV-2 RNA (red) in the lungs of infected K18-hACE2 mice. ISH was performed in a different mouse than that in Fig. 2. Cells were counterstained with hematoxylin.

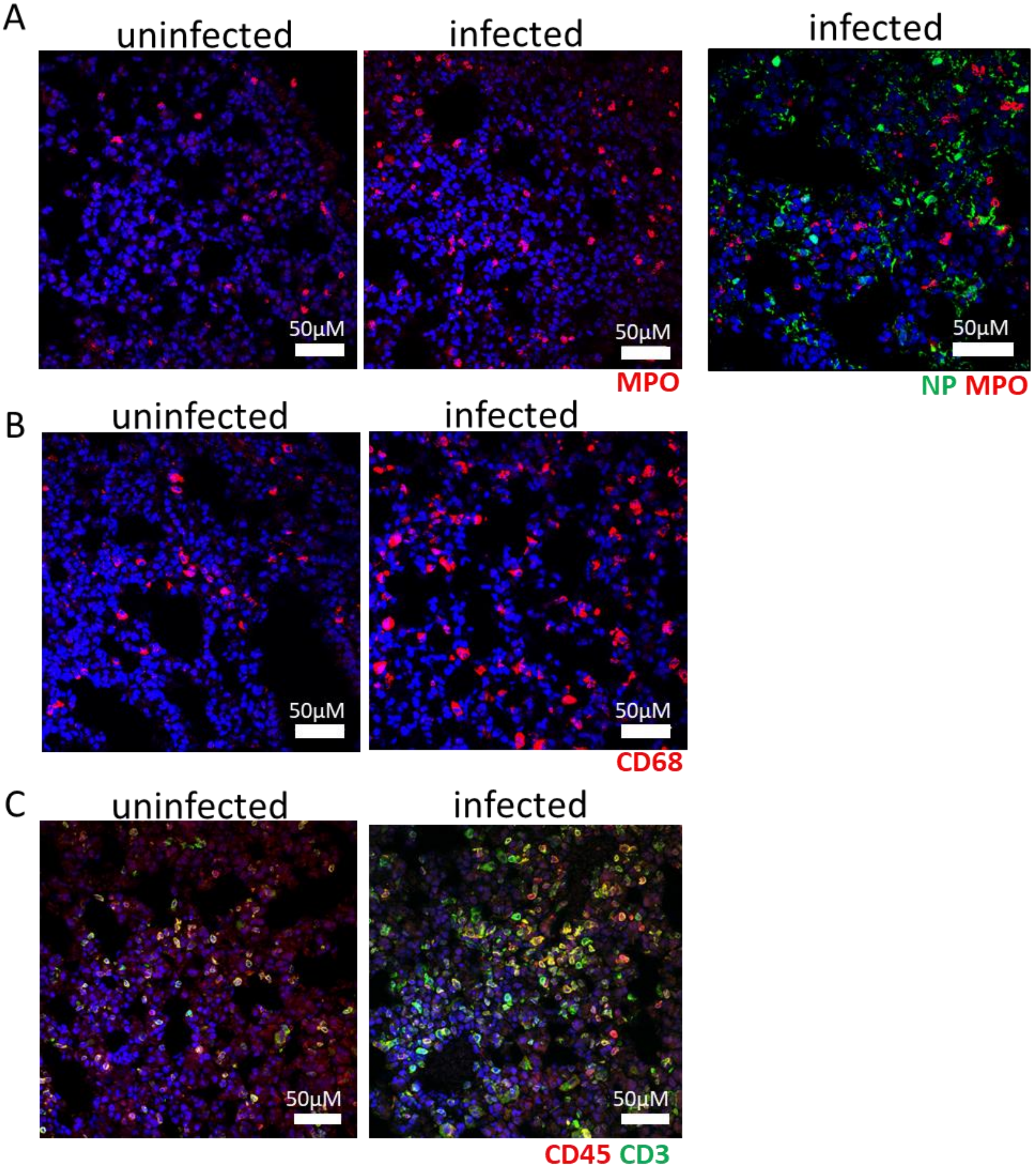

Figure S3. Infiltrating cells in the lungs of SARS-CoV-2 infected mice. A–C. IFA demonstrates increased number of myeloperoxidase (MPO)+ polymorphonuclear cells (neutrophils, eosinophils, and basophils) (A, red), CD68+ macrophages (B, red) CD45+ leukocytes (C, red) including CD3+ T cells (C, green) infiltrates in the lung of infected mice in comparison with the lung of uninfected mice. MPO positive cells (red) were devoid of viral NP protein (A, green). Nuclei are stained with DAPI (blue).

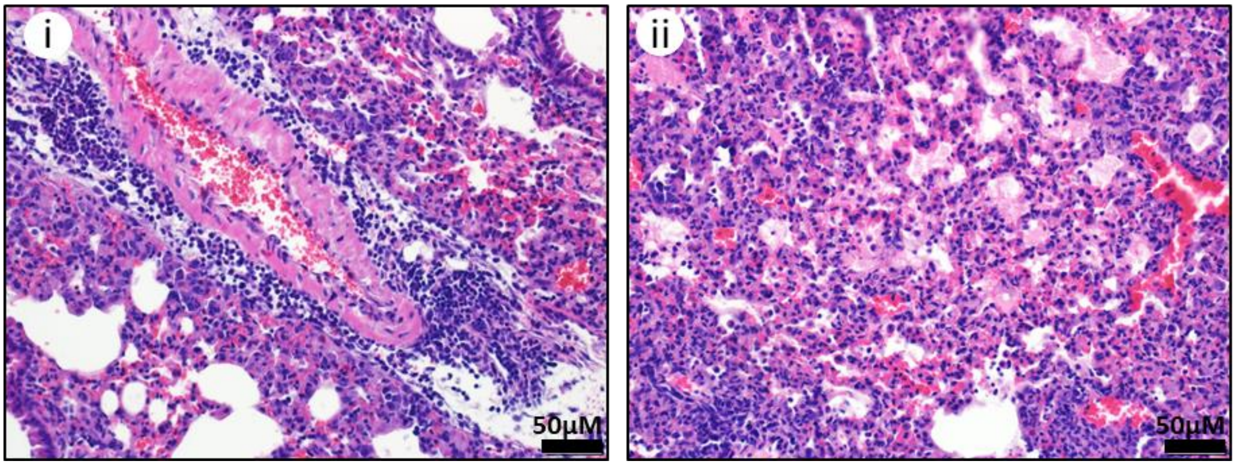

Figure S4. SARS-CoV-2 infection causes respiratory damage in K18-hACE2 mice. Representative H&E staining of lungs in infected K18-ACE2 mice. Edema, moderate numbers of mononuclear inflammatory cells, and fewer neutrophils expand the perivascular space surrounding an intermediate sized artery in the lung (i). Area of lung consolidation with inflammation/expansion of alveolar septa by fibrin, edema and mononuclear inflammatory cells; adjacent alveolar lumina are correspondingly filled with fibrin, edema and increased numbers of alveolar macrophages (ii).

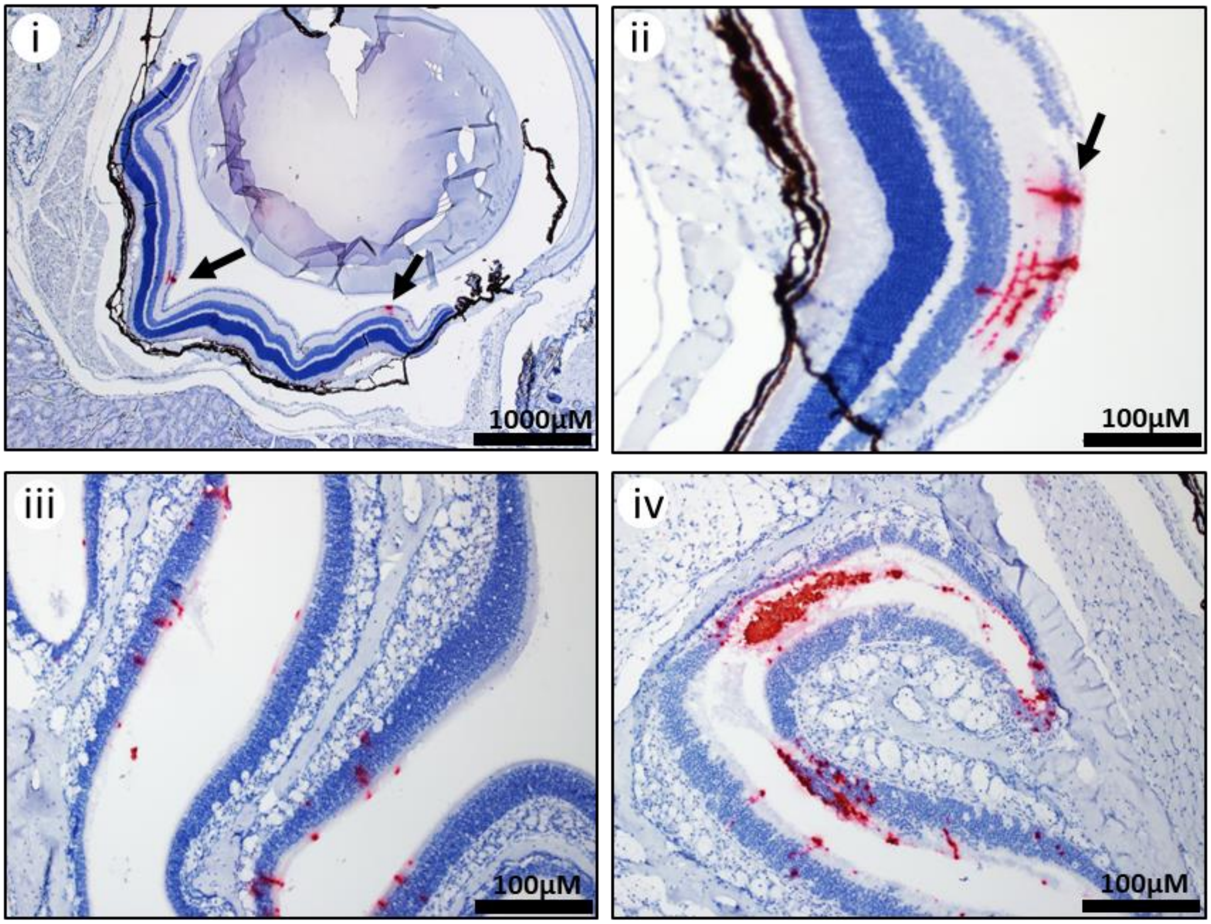

Figure S5. SARS-CoV-2 infection of eyes and nasal turbinates. Representative ISH staining showing the presence of SARS-CoV-2 RNA (red) in the eyes of infected K18-hACE2 mice with staining in the retina (arrows) (i & ii). Viral RNA was detected in the nasal turbiantes (iii & iv) with sloughing of infected cells (iv). Cells were counterstained with hematoxylin (blue).

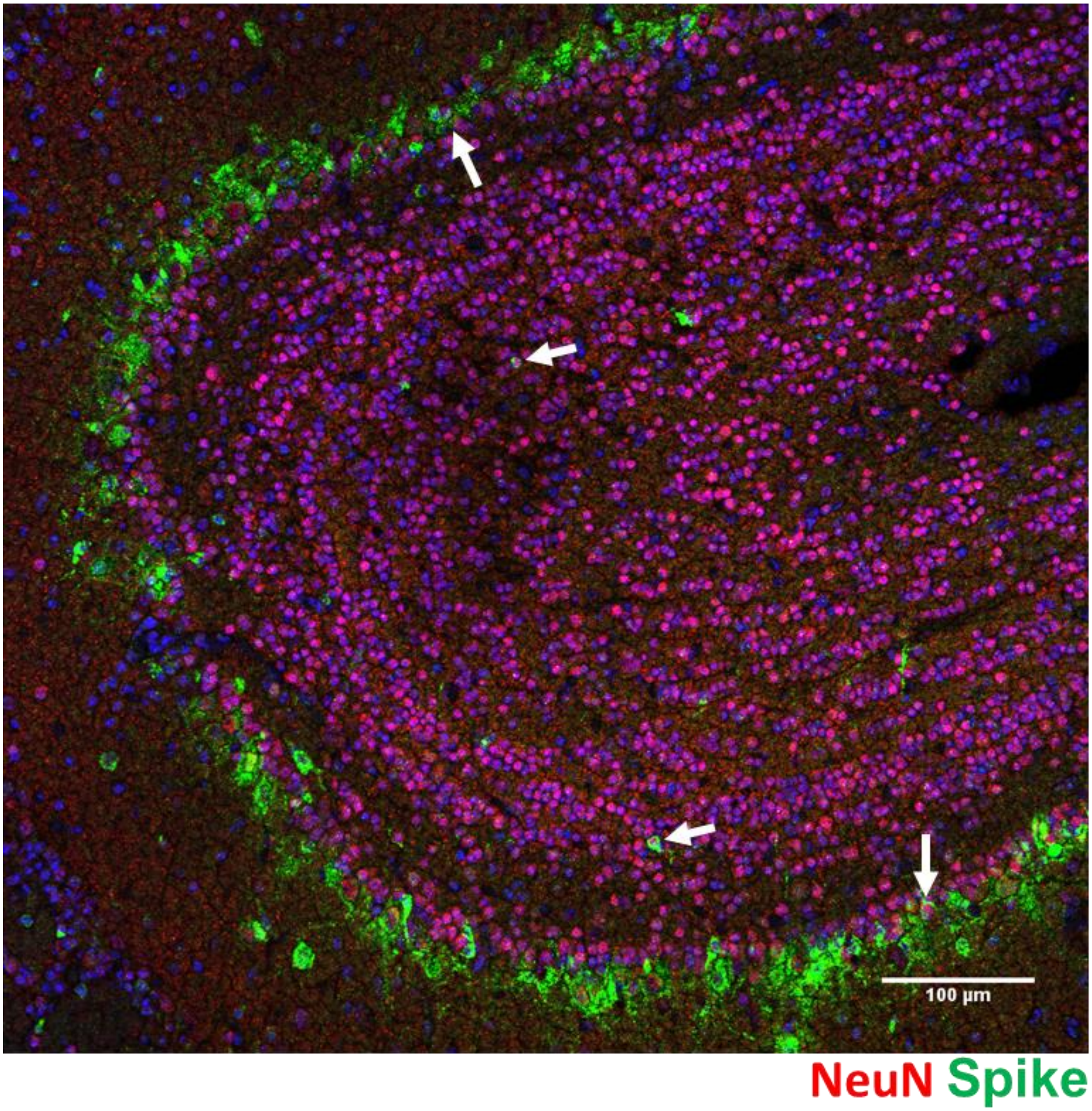

Figure S6. Infection of SARS-CoV-2 in the neurons of the olfactory bulb. Detection of viral spike protein (green) and the neuron marker NeuN (red) in infected olfactory bulb. Arrows denote co-stained cells. Nuclei are stained with DAPI (blue).

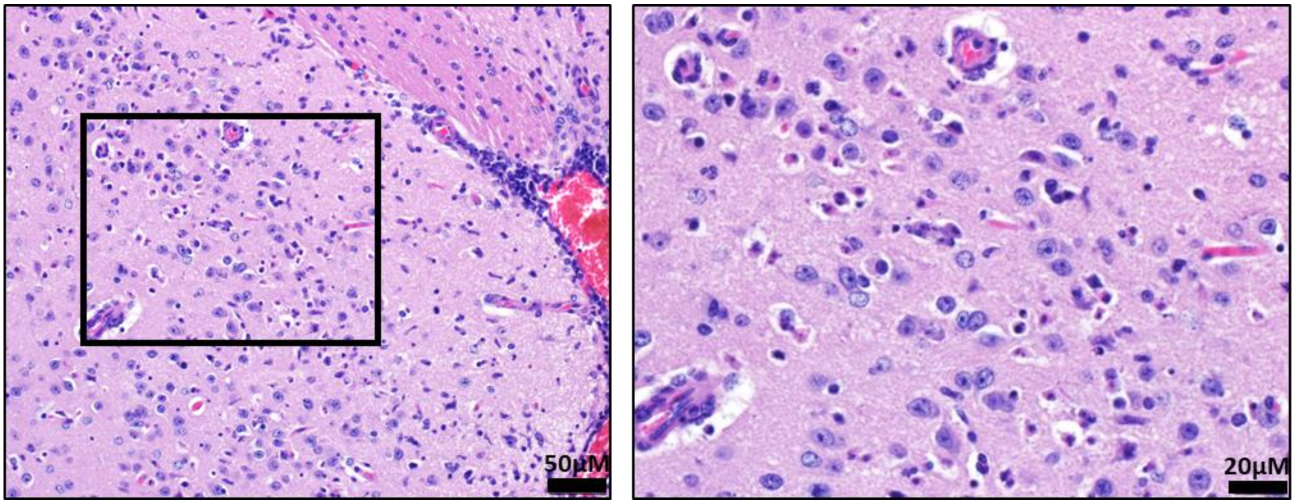

Figure S7. Brain lesions in SARS-CoV-2 infected mice. Representative H&E staining of lungs in infected K18-hACE2 mice. Multifocal areas of gliosis within the amygdala, predominantly characterized by increased numbers of microglia, and there are individual shrunken, angular cells with hypereosinophilic cytoplasm, pyknotic nuclei and surrounded by a clear halo, consistent with necrosis. While the morphology and location of individual necrotic cells is suggestive of neuronal necrosis, additional diagnostics are necessary to confirm the cell of origin. The right panel is a enhanced magnification of the boxed area

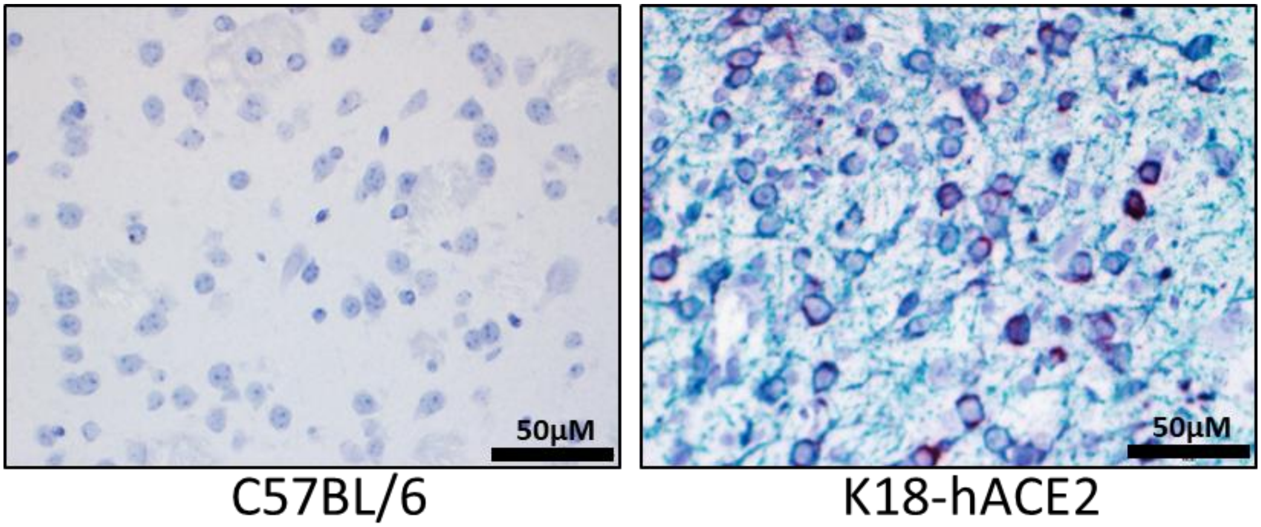

Figure S8. hACE2 transgene expression in neurons of SARS-CoV-2 infected mice. Representative ISH staining showing the presence of the hACE2 transgene (red) in infected K18-hACE2 mice. Duplex ISH staining further shows hACE2-exressping neurons are also positive to SARS-CoV-2 genomic RNA (green). Cells were counterstained with hematoxylin. C57BL/6 mice do not express the transgene.
